# Can we expect similar behavior among CuNiTi 35°C wires?

**DOI:** 10.1590/2177-6709.26.2.e211945.oar

**Published:** 2021-05-17

**Authors:** Ariane Salgado GONZAGA, David Salgado GONZAGA, Hallissa SIMPLÍCIO, Renato Parsekian MARTINS, Marília Regalado GALVÃO, Sergei Godeiro Fernandes Rabelo CALDAS

**Affiliations:** 1 Universidade Federal do Rio Grande do Norte, Departamento de Odontologia (Natal/RN, Brazil).; 2 Universidade Federal do Rio Grande do Norte, Departamento de Engenharia Mecânica (Natal/RN, Brazil).; 3 Universidade Estadual Paulista, Faculdade de Odontologia de Araraquara (Araraquara/SP, Brazil).

**Keywords:** Orthodontics, Orthodontic wires, Corrective orthodontics

## Abstract

**Objective:**

This paper aims to verify the thermodynamic, mechanical and chemical properties of CuNiTi 35ºC commercial wires.

**Methods:**

Forty pre-contoured copper-nickel-titanium thermodynamic 0.017 x 0.025-in archwires with an Af temperature of 35°C were used. Eight wires from five different manufacturers (American Orthodontics® [G1], Eurodonto® [G2], Morelli® [G3], Ormco® [G4] and Orthometric® [G5]) underwent cross-sectional dimension measurements, tensile tests, SEM-EDS and differential scanning calorimetry (DSC) tests. Parametric tests (One-way ANOVA and Tukey post-test) were used, with a significance level of 5%, and Pearson’s correlation coefficient test was performed between the Af and chemical elements of the wires. All sample tests and statistical analyses were double-blinded.

**Results:**

All wires presented standard dimensions (0.017 x 0.025-in) and superelastic behavior, with mean plateau forces of: G1 = 36.49N; G2 = 27.34N; G3 = 19.24 N; G4 = 37.54 N; and G5 = 17.87N. The Af means were: G1 = 29.40°C, G2 = 29.13°C and G3 = 31.43°C, with *p*>0.05 relative to each other. G4 (32.77°C) and G5 (35.17°C) presented statistically significant differences between each other and among the other groups. All samples presented Ni, Ti, Cu and Al in different concentrations.

**Conclusions:**

The chemical concentration of the elements that compose the alloy significantly influenced the thermodynamic and mechanical properties.

## RESUMO

**Objetivo:**

O presente artigo teve como objetivo verificar as propriedades termodinâmicas, mecânicas e químicas de fios CuNiTi 35°C comerciais.

**Métodos:**

Foram utilizados 40 arcos termodinâmicos pré-contornados de cobre-níquel-titânio de 0,017” x 0,025” e temperatura Af de 35°C. Oito fios de cinco fabricantes diferentes (American Orthodontics® [G1], Eurodonto® [G2], Morelli® [G3], Ormco® [G4] e Orthometric® [G5]) foram submetidos a medições de suas secções transversais, testes de tração, MEV-EDS e calorimetria diferencial (DSC). Foram utilizados testes paramétricos (One-way ANOVA e pós-teste de Tukey), com nível de significância de 5%, e foi realizado o teste do coeficiente de correlação de Pearson entre a temperatura Af e os elementos químicos dos fios. Todos os testes das amostras e análises estatísticas foram duplo-cegos.

**Resultados:**

Todos os fios apresentavam dimensões padronizadas (0,017” x 0,025”) e comportamento superelástico, com forças médias de platô de G1 = 36,49 N; G2 = 27,34 N; G3 = 19,24 N; G4 = 37,54 N; e G5 = 17,87 N. As médias de Af foram: G1 = 29,40°C, G2 = 29,13°C e G3 = 31,43°C, com *p*> 0,05 entre si. G4 (32,77°C) e G5 (35,17°C) apresentaram diferenças estatisticamente significativas entre si e entre os demais grupos. Todas as amostras apresentaram Ni, Ti, Cu e Al em diferentes concentrações.

**Conclusões:**

A concentração química dos elementos que compõem a liga influenciou significativamente as propriedades termodinâmicas e mecânicas.

## INTRODUCTION

Thermodynamic wires are wires that undergo changes in their crystallographic arrangement depending on the relationship between the temperature they are exposed to and their transition temperatures. These inherent temperatures can be manipulated by heat treatments or by atomic substitution, e. g. by replacing part of the nickel or titanium concentration of a nickel-titanium (Ni-Ti) alloy by copper (Cu), resulting in CuNiTi, a tertiary alloy.^1^ ^-^ ^6^ The addition of Cu to NiTi alloys also reduces stress and temperature hysteresis, giving more stability to the superelastic characteristics.^1^ ^-^ ^6^

Wires manufactured with this alloy have been marketed by Ormco Corporation^®^ under the name of “Copper NiTi”, with three austenite finish temperatures (Af) (27°C, 35°C and 40°C), enabling clinicians to quantify and apply appropriate levels of force for the orthodontic treatment.^1^ ^,^ ^2^ Now at the end of the patent term, several companies with different manufacturing processes, varying prices and possibly different quality manufacture these wires.

Although previous studies have shown a difference in the mechanical and thermodynamic behavior of these wires,^7^ ^-^ ^9^ the origin of these differences has not yet been explained. Thus, the present study analyzed the mechanical, thermodynamic and chemical characteristics of CuNiTi wires from five commercial brands in an attempt to show which wire characteristics are responsible for the particular behavior among brands of the same cross-sectional diameter. 

## MATERIAL AND METHODS

The sample consisted of forty 0.017 x 0.025-in pre-contoured CuNiTi wires, with austenitic finish (Af) temperature of 35°C, divided into five groups (G1 to G5), according to their commercial brand, for double-blinded tests (Table 1).

**Table 1:** Distribution of sample groups.

GROUP	NAME	BRAND	LOT
G1	Tanzo	American Orthodontics	C92395
G2	COBRE - NiTi	Eurodonto	F1408000
G3	Thermocopper NiTi	Morelli	2102848
G4	Copper NiTi 35°C	ORMCO	021544059
G5	FlexyNiTi Copper	Orthometric	020917001

All wires underwent cross-sectional measurements; differential scanning calorimetry (DSC); uniaxial tensile tests; scanning electron microscopy and energy dispersive spectroscopy (SEM/EDS). 

### CROSS-SECTIONAL MEASUREMENTS

The cross-sectional measurements of the wires were performed using a digital caliper with an accuracy of 0.001 mm (Starret, USA). Five wires randomly selected from each manufacturer were cleaned along with the caliper claws with alcohol, and each one was measured for both height and width at five different points of each archwire.^10^

### DIFFERENTIAL SCANNING CALORIMETRY (DSC)

To define the temperature transition range (TTR) of the wires, samples were taken from the straightest portion of each archwire. The samples were cut with orthodontic pliers into lengths of approximately 3 mm and weights of approximately 3.5 mg using a precision electronic scale with an accuracy of 10 µg.^11^ ^,^ ^12^ Each specimen was cleaned with alcohol, dried and placed in a covered and sealed aluminum crucible for a DSC test on a Netzsch Polyma DSC 214 instrument (Selb, Germany). An atmosphere of nitrogen gas at 50ml/min filled the heating chamber and an empty aluminum crucible was the inert reference.^13^ ^,^ ^14^

The temperature range of the test was from 60°C to - 40°C at 10°C/min. The DSC instrument was connected to Platinum software (TA Instruments, USA) to perform the analyses of the TTRs of exothermic and endothermic reactions, determining the initial and final temperatures of the phases.

### UNIAXIAL TENSILE TEST

The mechanical properties of the wires were defined by uniaxial tensile tests on a MultiTest 2.5d universal mechanical testing machine (Mecmesin Corporation, USA) with a 100 N loading cell (Mecmesin Corporation, USA) at the rate of 1mm/min for 2 mm of activation and deactivation. The length of the wire between the grips was 25 mm for all samples, measured with a 0.001-mm precision caliper (Starret^®^, USA). The samples did not reach the plastic deformation limit, as the deformation carried out was 8% of their initial length.^3^ ^,^ ^15^ ^,^ ^16^

**Figure 1:**
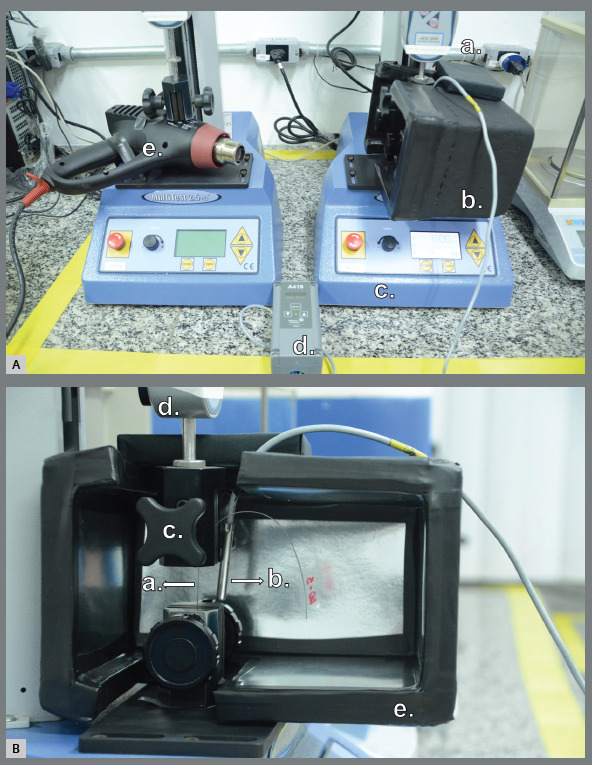
A) Frontal view of the setup of the tensile test with controlled temperature: a. load cell; b. styrofoam thermal box surrounding the entire clamp system; c. MultiTest 2.5d universal mechanical testing machine; d. temperature controller; e. hot air blower at minimum speed and distance of 30cm from the sample. B) View of the clamp system of the tensile test with controlled temperature: a. sample attached to the clamps; b. thermometer of the temperature controller placed behind the sample; c. clamp system; d. load cell; e. styrofoam thermal box surrounding the entire clamp system.

Samples damaged by crushing or sliding caused by the mechanical grips were discarded.^4^ The whole test was performed under a constant temperature of 37°C, maintained by a 1800 W hot air blower (Bosch^®^, Holland) at a minimum speed and a distance of 30 cm from the wires, linked to a temperature controller (Johnson Controls^®^ PENN, USA) and with a styrofoam thermal box surrounding the entire clamp system (Fig 1).

The midpoint of a linear regression line in the most horizontal segment of the load/deflection graph of the curves of deactivation in the martensitic phase determined the plateau of forces expressed by the wires, known as the superelastic clinical plateau, with a coefficient of determination of at least 0.99.^5^

The superelasticity rate (SE rate) was calculated using the ratio between the elastic modulus of the two lines generated in the deactivation curves of the tested wires (E2/E1). The first elastic modulus (E1) was obtained from the straight line of the superelastic clinical plateau, and the second (E2) was obtained from the first three points of the deactivation curve of the load/deflection graph. Wires presented superelastic behavior when their second SE rate was higher than 8.^5^ ^,^ ^17^

### ENERGY DISPERSIVE SPECTROSCOPY (SEM/EDS)

The microstructure of the wires was observed by SEM/EDS in a T-330 A JEOL-JSM microscope (Toronto Surplus & Scientific Inc., Canada)^18^ to determine the superficial chemical composition of the wires in each group and the phases (elements or associations) that compose them. A fractographic image was obtained and the chemical composition was automatically determined in percentages. 

### STATISTICAL ANALYSIS

Statistical analyses of the data were performed by comparing their normal distribution using the Kolmogorov-Smirnov test. ANOVA One-way and Tukey multiple comparisons tests were carried out considering a significance level of 5%. A Pearson correlation test was performed in order to investigate the correlation between the Af temperature and the chemical elements of the wires. All tests were done on SPSS^®^ software v.11.0 (IBM, USA) for Windows.

## RESULTS

### CROSS-SECTIONAL MEASUREMENTS

The cross-sectional dimensions were consistent with the information provided by the manufacturers (Table 2).

**Table 2:** Measurement of sample dimensions, in inches.

	n	HEIGHT		WIDTH	
		MEAN	SD	MEAN	SD
G1	5	0.017	0.00	0.025	0.00
G2	5	0.017	0.00	0.025	0.00
G3	5	0.017	0.00	0.025	0.00
G4	5	0.017	0.00	0.025	0.00
G5	5	0.017	0.00	0.025	0.00

### DIFFERENTIAL SCANNING CALORIMETRY (DSC)

The DSC tests showed that almost all wires presented an Af below that reported by the manufacturers, except group 5: G1) American Orthodontics^®^ = 29.40°C, G2) Eurodonto^®^= 29.13°C, G3) Morelli^®^= 31.43°C, G4) Ormco^®^= 32.77°C and G5) Orthometric^®^= 35.17°C (Table 3).

**Table 3:** One-way ANOVA and Tukey post-test for DSC tests results. Temperature values in °C.

	n	AS		PEAK A		AF	
		Mean	SD	Mean	SD	Mean	SD
G1	3	9.63^a^	0.42	19.80^a^	0.44	29.40^a^	0.62
G2	3	14.93^bc^	0.15	22.93^b^	0.25	29.13^a^	0.25
G3	3	10.10^a^	0.82	22.63^b^	1.02	31.43^a^	0.95
G4	3	12.90^a^	0.70	24.17^a^	0.85	32.77^b^	0.61
G5	3	17.00^bc^	1.93	27.53^b^	1.10	35.17^c^	0.85

### UNIAXIAL TENSILE TEST

The Uniaxial tensile tests showed that all wires presented superelastic behavior. The SE rate and mean plateau force were, respectively: 51.91 and 36.49 N for G1; 30.27 and 27.34 N for G2; 12.11 and 19.24 N for G3; 23.64 and 37.54 N for G4; 21.27 and 17.87 N for G5 (Table 4).

**Table 4:** Superelasticity rate (SE Rate) and plateau force in N.

	n	SE RATE		PLATEAU FORCE (N)	
		Mean	SD	Mean	SD
G1	5	51.91	61.62	36.49^b^	2.71
G2	5	30.27	16.29	27.34^ab^	12.54
G3	5	12.11	3.80	19.24^a^	8.36
G4	5	23.64	19.23	37.54^b^	2.61
G5	5	21.27	6.91	17.87^a^	5.02

### ENERGY DISPERSIVE SPECTROSCOPY (SEM/EDS)

**Figure 2:**
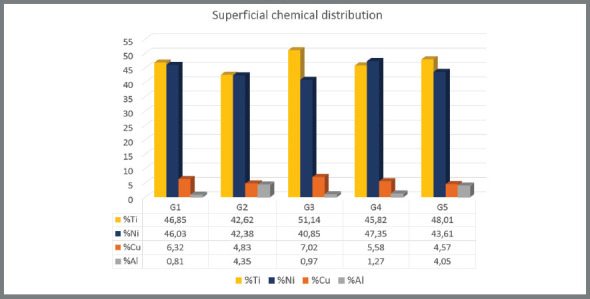
Graph of the surface concentration of the chemical elements of the alloys of each manufacturer.

The percentage of titanium (Ti), nickel (Ni), copper (Cu) and aluminum (Al) surface concentrations for the archwires of each manufacturer is given in Figure 2. The Pearson correlation test performed between Af and the chemical elements was not significant (*p* > 0.05).

## DISCUSSION

When an orthodontic wire with superelastic characteristics is used during orthodontic treatment, the force expressed by this wire on a tooth depends on various factors. These include the temperature at which the transformation of martensite to austenite begins until it reaches the complete transformation (Af) the amount of deformation applied during the activation of this wire, and also the manufacturing processes of the material.^6^ Thus, the precise development of variations in the transition temperatures of each wire is one of the essential factors for them to behave clinically as expected, expressing their characteristics of superelasticity and pseudoelasticity. However, if variations in the TTR of the wires due to the manufacturing technique exist, it is possible that the wires produced by different manufacturers express different thermodynamic and mechanical behaviors.^6^ ^-^ ^8^

**Figure 3:**
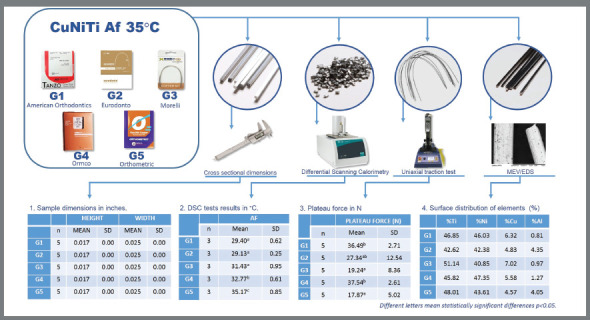
Graphic summary of materials and methods, and results.

Several previous works have already shown that differences among the mechanical behavior of thermoactive archwires of diverse manufacturers and interlot variations are a fact.^4^ ^,^ ^5^ ^,^ ^7^ ^-^ ^9^ The novelty of this study lies in its questioning of what variable in the alloy is responsible for the diverse mechanical behavior of the wires that should theoretically express the same patterns (Fig 3).

In order to understand any discrepancies between the wires of different manufacturers analyzed in this study, height and width of each wire were measured to verify the existence of any dimensional differences. All groups showed standard dimensions (0.017 x 0.025-in), and therefore this factor was excluded as a possible explanation for any behavioral variations.

Among the brands evaluated, only Orthometric^®^ (G5) FlexyNiTi Copper wire reached the Af temperature reported by the manufacturer (35°C), with a statistically significant difference to the other groups. G5 also presented the lowest mean plateau force (17.9N) at deactivation. Therefore, at an oral temperature of 37°C, the wire is expected to be at its final austenitic phase. However, due to the stress-induced martensitic (SIM) transformation during clinical installation, the wire will be expressing its pseudoelasticity. This fact implies the transfer of lower and constant forces during the orthodontic treatment, which is the ideal situation for tooth movement.^1^ ^-^ ^4^ ^,^ ^6^ ^,^ ^18^ ^,^ ^19^ ^,^ ^20^ ^-^ ^23^

This thermal and mechanical behavior can be explained by the chemical composition of the alloy. The concentration of Ti was higher than that of Ni (48.01% and 43.61%), and this difference was the second largest among the evaluated brands. Furthermore, the addition of Cu becomes essential so that the wire has a suitable and stable transition temperature for clinical use.^5^ ^,^ ^6^ ^,^ ^19^ ^,^ ^23^ ^,^ ^24^

The results showed that the Cu concentration was 4.57% in G5, which was the lowest of all. This low concentration was not expected to present satisfactory results in relation to Af compared to the copper concentrations in the other brands. However, the Al concentration was 4.05%, which was much higher than the other brands, except for G2. This suggest that the association of these chemical elements may have been responsible for the excellent thermodynamic behavior of this wire. 

The Pearson correlation test showed that, although weak, there was a negative correlation between Af and Cu (ρ = -0.408), and a positive correlation between Af and Al (ρ = 0.168). This could mean that a higher concentration of Cu in the alloy tends to decrease Af, while the higher concentration of Al in the alloy tends to increase Af, corroborating with the interpretations obtained from the results and other reports.^7^ ^,^ ^8^ ^,^ ^9^ ^,^ ^25^

The Eurodonto^®^ COBRE NiTi wires (G2) showed similar concentrations of Cu and Al (4.83% and 4.35%, respectively); however, their thermal behavior was lower (Af = 29.13°C). In addition, it showed an equiatomic ratio between nickel and titanium (Ni 42.38% and Ti 42.62%), and a plateau force of 27.3N. The fact that there is a balanced ratio between nickel and titanium in these wires already guarantees stability in the thermal and mechanical behavior of this material,^5^ ^,^ ^24^ and no specific treatment is required to prevent decomposition at other phases of intermediate temperatures^5^. Therefore, it seems safe to say that the addition of copper to the alloy together with aluminum caused a decrease in the variation of the final austenitic temperature, justifying the thermodynamic behavior of these wires.^5^ ^,^ ^6^ ^,^ ^19^ ^,^ ^23^ ^-^ ^25^

The Tanzo wires from American Orthodontics^®^ (G1) reached a mean high-plateau force of 36.49 N and average Af temperature of 29.4°C; below that identified on their packaging. Similar to the Eurodonto^®^ wires (G2), the SEM-EDS test in this metal alloy showed a superficial chemical composition in which the concentration of Ni and Ti is balanced (46.03% and 46.85%); however, with a Cu concentration equal to 6.32% and a smaller quantity of Al (0.81%). The fact that the Af temperature in this group is practically 7°C below the mean body temperature suggests that the crystalline structure of this metal alloy reached its fully stabilized austenitic phase when inserted into the intraoral environment.^3^ ^,^ ^5^ ^,^ ^9^ ^,^ ^22^ ^,^ ^25^ In the case of this study, this occurred when subjected to a uniaxial traction test with a controlled temperature of 37°C. Thus, apparently the deformation levels required for transforming the final austenitic phase in stress-induced martensitic becomes higher. Therefore, the deformation applied in the study might not have been sufficient for the complete transformation phase to occur, resulting in high values in the tensile test.^1^ ^-^ ^4^ ^,^ ^6^ ^,^ ^18^ ^,^ ^20^ ^-^ ^23^

The Morelli^®^ THERMOCOPPER NiTi orthodontic wire samples (G3) presented the second lowest mean plateau force (19.2 N) and an Af temperature of 31.43°C. Its chemical composition revealed the highest concentration of Ti and Cu compared to all groups (51.14% and 7.02%), the lowest concentration of Ni (40.85%) and an Al concentration of 0.97%. The discrepancy between Ni and Ti concentration could generate a thermal behavior with significant variations in Af temperatures. However, the high concentration of Cu in the alloy stabilizes the Af temperature at a value close to that indicated by the manufacturer. This result corroborates with the literature that states that the addition of Cu has the ability to stabilize the TTR, making the thermodynamic behavior less sensitive to Ni and Ti concentrations.^5^ ^,^ ^6^ ^,^ ^19^ ^,^ ^23^ ^-^ ^25^ If the Cu concentration was a little lower in this material and the Al concentration was higher, its Af temperature would probably be closer to 35°C. Such changes would tend to increase the final austenitic temperature value for this wire due to the negative correlation of Af with Cu and the positive correlation with Al. Although these wires have Af temperatures lower than the information provided on their packaging, their mechanical properties were statistically classified as similar to the G5 group. Therefore, their mechanical properties along with their low mean plateau force makes them suitable for clinical use within the specific indications for the use of thermodynamic wires at 35°C .

The evaluations of the Copper Ni-Ti ORMCO^®^ wires (G4) revealed an Af temperature of 32.77°C for this group; this was the second highest of the brands studied, and the highest plateau force average (37.5 N) of all the wires evaluated. In addition to these parameters, G4 was also the only group with a Ni concentration (47.35%, highest of all samples) higher than Ti (45.82%), and it had a Cu concentration of 5.58% and Al equal to 1.27%. Thus, the Cu makes the Af temperature closer to that indicated by the manufacturer.^5,6,19,23-25^ However, the low Al concentration, and a relatively low concentration of Ti , which are elements that correlate positively with Af, in addition to the high concentration of Ni, which has a negative correlation with Af, are probably the reasons that this sample expressed high plateau forces and its Af temperature was below 35°C. 

Among the results found in this study, the one not expected was the inclusion of aluminum in the alloy, since the manufacturers did not include it in the description of the compositions, naming all wires of this kind as a tertiary alloy - CuNiTi. Nowadays, NiTi and Cu-based alloys are the most studied shape memory alloys (SMAs) in this class of materials, due to their unique thermomechanical behavior. However, despite the preference for NiTi, which is currently the most used SMA, Cu-based SMAs are emerging as potential substitutes, mainly in metallurgical studies for diverse uses, in particular, superelastic Cu_17_Al_11_Mn_4_ that exhibits mechanical properties that are similar to those of NiTi, but less expensive.^25^ Although the literature has shown that binary and tertiary alloys containing Cu and Al in their composition have exhibited excellent thermal and electrical properties, as well as shape memory and superelasticity,^5^ ^,^ ^25^ this information has not been reported in orthodontic wires yet.

Despite the differences found in this study, all the wires evaluated can be used clinically, however, the thermodynamic properties must be considered, in order to enhance the mechanical potential of the wires and optimize the clinical outcomes. Wires with a higher Af temperature demand minor activations to reach the superelastic plateau at deactivation when exposed to body temperature during clinical use, while under the same environmental conditions, those wires with a lower Af temperature need higher activations to express the superelastic plateau at deactivation.

## CONCLUSION

According to the results obtained in the study, we can conclude that:

» The chemical concentration of the elements that compose the alloy significantly influenced the thermodynamic and mechanical properties.
» In nickel and titanium equiatomic alloys (G2 and G3), the addition of Cu and Al reduces the TTR, while in non-equiatomic alloys (G1, G4 and G5), the addition of these elements increases the TTR, which is reflected in their Af temperatures.
» Aluminum seems to play a fundamental role in increasing the TTR when the copper concentration is low (G5).
» In the alloys with higher concentrations of Ti (G3 and G5), the deactivation force levels were lower, while in the alloys with higher concentrations of nickel (G4) the deactivation force levels were higher.
» The addition of copper and aluminum to the alloy should be to substitute nickel rather than titanium, to ensure lower levels of deactivation forces.
